# Relationship Between Metabolites of Vitamin D, Free 25-(OH)D, and Physical Performance in Indoor and Outdoor Athletes

**DOI:** 10.3389/fphys.2022.909086

**Published:** 2022-07-08

**Authors:** Anna Książek, Aleksandra Zagrodna, Małgorzata Słowińska-Lisowska, Giovanni Lombardi

**Affiliations:** ^1^ Department of Biological and Medical Basis of Sport, Faculty of Physical Education and Sports, Wroclaw University of Health and Sport Sciences, Wroclaw, Poland; ^2^ Laboratory of Experimental Biochemistry & Molecular Biology, I.R.C.C.S. Istituto Ortopedico Galeazzi, Milano, Italy; ^3^ Department of Athletics, Strength and Conditioning, Poznań University of Physical Education, Poznań, Poland

**Keywords:** vitamin D, 24,25-(OH)2D3, 3-epi-25-(OH)D, 1,25(OH)2D, VDBP, vertical jump, physical performance

## Abstract

The potential effects of vitamin D in athletes have received considerable attention in the literature. However, little is known about vitamin D metabolites and their association with physical performance in athletes. Therefore, the aim of our study was to determine the relationship between metabolites of vitamin D, vitamin D binding protein (VDBP), free, bioavailable 25-(OH)D, and physical fitness tests in athletes. A total of 40 indoor and outdoor players (16 judoists and 24 football players) participated in the study. Vitamin D metabolites (25-(OH)D, 24,25-(OH)_2_D_3_, 3-epi-25-(OH)D_3_, and 1,25-(OH)_2_D) were assessed using LM-MS/MS. Free 25-(OH)D concentration was evaluated by calculation using serum albumin and VDBP levels. Athletic performance was assessed using handgrip and vertical jump. Our study showed a significant correlation between vitamin D metabolites and handgrip strength and vertical jump variables in indoor players. It demonstrated a significant association between 3-epi-25-(OH)D_3_ and vertical jump parameters in outdoor players. The results of our study showed relationship between free, bioavailable 25-(OH)D, and vertical jump variables in indoor players. In conclusion, we provide novel information on the vitamin D metabolites and athletic performance in athletes. Based on the results of our study, we concluded that vitamin D metabolites might be involved in skeletal muscle function in relation to athletic performance.

## 1 Introduction

During the last few decades, interest has increased in vitamin D research because studies frequently evidence vitamin D deficiency/insufficiency among athletes worldwide (Valtueña et al., 2014; Krzywanski et al., 2016; Lombardi et al., 2017). Findings from a meta-analysis of 23 studies, on a total of 2,313 athletes, demonstrated that 56% of those practicing both indoor and outdoor activities had insufficient vitamin D concentrations (Farrokhyar et al., 2015). Further, for athletes living at latitudes above 40°N, the risk of vitamin D inadequacy significantly increases during wintertime (Farrokhyar et al., 2015).

Current studies indicate that vitamin D plays a role in skeletal muscle function. Calcitriol [1α,25-(OH)_2_D] stimulates the myogenic differentiation of mesenchymal stem cells and upregulates the insulin growth factor (IGF)-II and follistatin expression (Garcia et al., 2011). It downregulates, instead, IGF-I and myostatin protein expression (Di Luigi et al., 2020). 1α,25-(OH)_2_D can enhance the sensitization of calcium binding sites at the sarcoplasmic reticulum level (Girgis et al., 2013) and influence muscle cell growth and differentiation, particularly of the fast-twitch type II fibers (Halfon et al., 2015). In addition, vitamin D increases insulin sensitivity, energetic substrate metabolism, and oxidative capacity by regulating mitochondrial respiration (Antinozzi et al., 2017; Ashcroft et al., 2020). 1α,25-(OH)_2_D exerts different non-classic biological effects that are supposed to influence health status, exercise, and sport performance in athletes (Owens et al., 2018).

Recent advances in mass spectrometry (MS)-based detection of plasma metabolites have allowed for the accurate detection of 1,25-(OH)_2_D, 24,25-(OH)_2_D, a major catabolic metabolite of 25-(OH)D, and 3-epi-25-(OH)D. Although only calcitriol has defined biological activities, there is interest in the other vitamin D metabolites. For instance, studies involving animal models demonstrated that 24,25-(OH)_2_D exerts a crucial role in the maintenance of bone integrity, healing, and function (Seo et al., 1997; Seo and Norman, 1997). Furthermore, 24,25-(OH)_2_D modulates growth plate chondrocyte physiology and parathyroid gland function (Herrmann et al., 2017). In turn, 3-epi-25-(OH)D levels are directly associated with the cardiovascular risk profile, and 3-epi-1α,25(OH)_2_D, a derivative of 3-epi-25-(OH)D, effectively reduces blood parathyroid hormone (PTH) without affecting calcium levels (Brown et al., 2005; Lutsey et al., 2015). To our knowledge, there are only few studies, especially intervention ones, related to the status of different vitamin D metabolites (Hassan-Smith et al., 2017; Mieszkowski et al., 2020; Mieszkowski et al., 2021).

Circulating levels of calcidiol [25-(OH)D] are currently used as the indicator of vitamin D status (Bikle et al., 2017; Tsuprykov et al., 2018). Recently, there has been speculation as to whether just measuring total 25-(OH)D is appropriate for the assessment of vitamin D status in different physiological and pathophysiological conditions (Bikle et al., 2017). Around 85%–90% of the total circulating 25-(OH)D and 1α,25-(OH)_2_D is bound to vitamin D binding protein (VDBP). About 10%–15% is bound to albumin, in contrast to free 25-(OH)D [bioavailable 25-(OH)D], which accounts <1% of total circulating vitamin D (Bikle et al., 2017; Owens et al., 2018). According to the “free hormone” hypothesis (Chun et al., 2014), current studies have shown that only the non-bound fraction of vitamin D is able to enter cells and to exert biologic effects (Bikle and Schwartz, 2019). It has been documented that the bioavailable fraction of circulating 25-(OH)D displays a stronger association with bone mineral density (BMD) than total levels, in healthy adults (Powe et al., 2011), and with intact PTH levels (iPTH) (Shieh et al., 2016).

More recently, the relationships between total 25-(OH)D concentration and performance-related factors in athletes have been observed in many studies (Seo et al., 2019; Kim et al., 2020; Most et al., 2021; Wilson-Barnes et al., 2021). Although the importance of vitamin D for athletes in regard to health and performance was emphasized, the studies found inconsistent results. A main issue is represented by the fact that those studies did not consider the metabolites of vitamin D and the amount of free or bioavailable 25-(OH)D, important to muscle function. Considering the role of vitamin D in skeletal muscle and athletic performance, there is a need to assess such factors among this population. Therefore, the primary aim of our study was to assess circulating vitamin D metabolites, VDBP, free, bioavailable 25-(OH)D concentration in athletes by considering the behavior of their training, either indoor or outdoor. A secondary aim was to determine the relationship between metabolites of vitamin D, VDBP, free, bioavailable 25-(OH)D, and physical fitness tests in athletes.

## 2 Methods

### 2.1 Participants

Forty injury-free male athletes (16 judoists and 24 football players) were included in the study. All participants were Caucasians with white skin, and none of them was a regular sunbed user. None of the subjects used any food supplements containing vitamin D and calcium. Participants’ characteristics are shown in Table 1. The study was carried out in Wrocław (Poland), which is situated at the latitude of 51°10’ N. It was conducted during the general preparatory period in the 2^nd^ Polish national football team and academic judo team. Athletes within sport discipline had similar exercise loads. Data were collected during 2 weeks at the turn of February and March 2021.

**TABLE 1:** Athletes’ characteristic and physical performance parameters.

	Mean ± SD			
	Judoists (*n* = 16)	Football Players (*n* = 24)	*p*-value	Cohen’s d
Age (years)	22.5 ± 2.9	21.9 ± 3.7	0.56	−0.18
Body weight (kg)	82.1 ± 10.8	78.3 ± 6.2	0.21	−0.46
Height (cm)	181.4 ± 5.0	182.0 ± 5.6	0.71	0.12
Body fat (%)	11.3 ± 2.5	10.8 ± 1.4	0.46	−0.27
Career duration (years)	12.3 ± 1.0	12.4 ± 1.0	0.56	0.19
Training time (h/week)	15.1 ± 1.4	11.0 ± 1.1	**<0.001**	−3.4
Calcium (mg/dL)	9.97 ± 0.34	9.90 ± 0.44	0.57	−0.18
ACa (mg/dL)	9.36 ± 0.28	9.34 ± 0.36	0.78	−0.08
iPTH (pg/mL)	50.3 ± 17.2	40.2 ± 19.6	0.09	−0.54
Albumin (g/dL)				
Total 25-(OH)D [ng/mL)	19.96 ± 9.31	22.86 ± 8.33	0.32	0.33
25-(OH)D_3_ (ng/mL)	19.31 ± 9.27	22.0 ± 8.52	0.36	0.30
25-(OH)D_2_ (ng/mL)	0.65 ± 0.35	0.86 ± 0.47	0.12	0.49
24,25-(OH)_2_D_3_ (ng/mL)	1.30 ± 0.91	1.51 ± 0.98	0.49	0.22
3-epi-25-(OH)D_3_ (ng/mL)	0.72 ± 0.52	0.92 ± 0.51	0.23	0.39
1,25-(OH)_2_D (pg/mL)	44.69 ± 25.44	40.40 ± 26.97	0.61	−0.16
VDBP (µmol/L)	2.25 ± 0.41	2.48 ± 0.52	0.14	0.47
Free 25-(OH)D M1 (pg/mL)	12.71 ± 6.11	14.07 ± 6.09	0.50	0.22
Free 25-(OH)D M2 (pg/mL)	10.20 ± 4.91	11.45 ± 4.81	0.49	0.22
Bioavailable 25-(OH)D (ng/mL)	4.23 ± 2.04	4.70 ± 1.98	0.54	0.20
Handgrip (kg)				
L	49.6 ± 11.4	41.3 ± 7.5	**0.018**	−0.89
R	48.8 ± 12.1	44.5 ± 7.4	0.27	-0.40
Jump height (m)	0.38 ± 0.06	0.38 ± 0.04	0.85	−0.07
Peak force (N)	2,173 ± 321	2,017 ± 209	0.10	−0.59
Peak velocity (m/s)	2.85 ± 0.21	2.83 ± 0.14	0.81	−0.09
Peak power (W)	4,535 ± 919	4,289 ± 515	0.35	−0.34
RSImod (m/s)	0.48 ± 0.08	0.47 ± 0.06	0.77	−0.10
Peak force (N/kg)	26.2 ± 1.9	25.6 ± 2.0	0.37	−0.31
Peak power (W/kg)	54.4 ± 6.0	54.4 ± 5.3	0.98	0.01
Peak propulsive force/kg (N/kg)	26.1 ± 1.9	25.6 ± 1.9	0.39	−0.29
AVG propulsive force/kg (N/kg)	20.8 ± 1.2	20.7 ± 1.4	0.94	−0.03
Peak propulsive power/kg (W/kg)	54.2 ± 6.1	54.5 ± 5.3	0.87	0.06
AVG propulsive power/kg (W/kg)	31.5 ± 3.3	31.0 ± 2.7	0.58	−0.19
VO_2max_ (ml/kg/min)	50.9 ± 2.1	53.4 ± 3.5	**0.02**	0.62

Bold values are statistically significant. iPTH, intact parathyroid hormone; L, left; R, right; RSImod, reactive strength index—modified; VDBP, vitamin D binding protein; AVG, average; VO_2max_, maximal oxygen uptake.

Measurements were performed under the fasting state. Height was measured by an anthropometer accurate to up to 1·10^–3^ m (GPM Siber Hegner Machinery Ltd., Zurich, Switzerland). The body mass was measured with the use of an electronic scale accurate up to 10^2^ g (Fawag, Lublin, Poland).

Skinfold thickness was measured at seven sites (biceps, triceps, subscapular, suprailiac, abdominal, thigh, and calf) with a Harpenden skinfold calliper (British Indicators, Burgess Hill, United Kingdom). Each measurement was taken by the same person, 3 times, and the mean value was used for calculation. Percentage body fat was assessed using the formula (Withers et al., 1987; Siri, 1993).

### 2.2 Biochemical Analyses

Athletes were instructed to visit laboratory between 7.00 and 10.00 a.m., after 10-to-12-h fasting and 24-h abstinence from training. Blood samples were collected into plain tubes, containing a clot activator (Vacutest, Kima, Italy). Blood was kept at room temperature for 1 h and then centrifuged at 1300 *g*, for 10 min, at 22°C. Serum was aliquoted and stored at −80°C until assayed.

iPTH in blood serum was determined by the electro-chemiluminescence assay (ECLIA) on an Elecsys analyzer (Roche, Switzerland) according to the manufacturer’s protocol and specific assay laboratory quality control procedures. The intra- and inter-assay coefficients of variation (CVs) were 4.5% and 4.8%, respectively, and the limit of detection was at 1.20 pg/mL (0.127 pmol/L).

Total calcium (Ca) was determined, in serum, by a colorimetric assay using the Konelab 60 system from bioMérieux (France).

Albumin was assayed on a Siemens Dimension Xpand Plus clinical chemistry system (Siemens, Germany).

Albumin-adjusted calcium (*ACa*) was calculated using the formula

$$ACa\;=Ca\;+\left[\left(4-albumin\;\left(\frac{g}{dL}\right)\right)*\;0.8\right].$$

### 2.3 Vitamin D Metabolite Levels

The vitamin D metabolite [25-(OH)D_3_, 25-(OH)D_2_, 24,25-(OH)_2_D_3_, 3-epi-25-(OH)D_3_, and 1,25-(OH)_2_D_3_] levels were determined by quantitative analysis *via* liquid chromatography coupled with tandem mass spectrometry (LC-MS/MS; QTRAP^®^4,500 (Sciex) coupled with an ExionLC HPLC system), with minor changes according to a previously published method (Rola et al., 2020).

### 2.4 Free Vitamin D *via* Vitamin D Binding Protein

VDBP was measured by the enzyme-linked immunosorbent assay (ELISA, R and D Systems, Minneapolis, MN, United States) according to the manufacturer’s instructions. The intra-assay CV ranges between 5% and 7%, and the inter-assay CV ranges between 5% and 8%.

The levels of free 25-(OH)D were calculated from total measured 25-(OH)D, VDBP, and serum albumin concentrations by using the equations by Bikle et al. (1986) (Method 1—M1),

$$Free\;25-\left(OH\right)D=\frac{total\;25-\left(OH\right)D}{1\;+\;\left(6\;x\;10^{3}x\;albumin\right)+\left(7\;x\;10^{8}\;x\;VDBP\right)}.$$

Moreover, free, bioavailable 25-(OH)D concentration was calculated using equations adapted from those described by Vermeulen et al. (1999) (Method 2—M2). Vermeulen equations were adapted by replacing the variables for testosterone, SHBG, and albumin and their respective binding constants with those of 25-(OH)D, VDBP, and albumin,

$$FT=\frac{\left(\left[T\right]-\left(N\;x\;\left[FT\right]\right)\right)}{\left(K_{t\;}\left\{SHBG-\left[T\right]+N\left[FT\right]\right\}\right)},$$

where *K*_*t*_ is the association constant of SHBG for *T* and *N=KaCa+1*. This yields a second degree equation that can be solved for either FT or SHBG (Vermeulen et al., 1999).

### 2.5 Physical Fitness Tests

#### 2.5.1 Hand Grip Strength

Hand grip strength was measured by a manual dynamometer (T.K.K.5001, Takei Scientific Inst. Co., Ltd., Niigata, Japan) at a resolution of 10^2^ g and an accuracy of 5·10^2^ g. Prior to testing, each subject was instructed on the correct performance of the measurements. Each subject was asked to comfortably hold the dynamometer with their fingers and palm tight on the device. They then lowered their upper limb along the trunk, while keeping a certain distance, so that neither the elbow nor the hand touched the body, and gripped the dynamometer using maximum muscle power. Throughout the test, subjects were asked to stand with their feet apart and the other upper limb freely along the body. Measurements were taken in kilograms (kg).

#### 2.5.2 Vertical Jump

The maximum power, force, velocity, reactive strength index—modified (RSImod), and propulsive force/power of the lower limbs were determined by the vertical jump test using a dual single-axis force platform system (Pasco PS 2141 plates, Pasco Scientific Inc., Roseville, CA, United States) with a sampling frequency of 1,000 Hz, unfiltered (Owen et al., 2014). Participants were instructed to stand with one foot on each platform with their hands on their hips. Athletes were asked to stand still for a period of 3 s to allow the system to ascertain body weight and the onset of movement threshold (Owen et al., 2014; McMahon et al., 2018). Once the system had recorded body weight, the participant was instructed to jump as high as possible while keeping their hands on their hips. For each jump, the athlete was required to return to the “quiet standing” position with the hands remaining on the hips. Three attempts were given for each athlete.

#### 2.5.3 Aerobic Fitness

Aerobic fitness was determined using standardized 30–15 intermittent fitness test (30-15IFT). The test consisted of 30 s of shuttle runs with 15 s of passive recovery (Buchheit, 2008). The test starting velocity was set at 8 km/h, and the speed was increased by 0.5 km/h every 30 s. Participants run back and forth between two lines (40 m apart) at a pace directed by a pre-recorded audible signal. Subjects ran until voluntary exhaustion or when subjects could no longer maintain the required running speed were asked to stop the test. The distance covered at that point was recorded as the test result.

### 2.6 Statistical Analysis

Descriptive statistics were presented using mean standard deviations. The normality of data was assessed using the Shapiro–Wilk test and homogeneity of variance using the Levene test. The difference in athletes’ characteristic and physical performance tests between both groups of athletes was analyzed using the Welch two sample *t*-test.

In addition, the effect size was determined by Cohen’s d with 95% confidence interval. The effect size was defined as small if < 0.2, medium if between 0.2 and 0.5, large if between 0.5 and 0.8, and very large if > 0.8.

Multiple regression was performed to analyze association between iPTH, Ca, ACa, and vitamin D metabolites levels. Moreover, we used this analysis to check relationship between vitamin D metabolites levels and athletic performance. Statistically significant models were adjusted for age, body mass, and body fat.

All analyses were performed with R for Windows, version 4.1 (R Foundation for Statistical Computing, Vienna, Austria). *p* < 0.05 was selected as the significance threshold.

## 3 Results

We included forty participants in this study. Athletes competed in two sport’s discipline: judo (classified as indoor) and football (classified as outdoor) (Table 1). Indoor athletes’ weekly training hours were significantly higher than outdoor (15.1 ± 1.4 vs. 11.0 ± 1.1 h, *p* < 0.001). VO_2max_ was significantly higher in outdoor than in indoor players (53.4 ± 3.5 vs. 50.9 ± 2.1 ml/kg/min, *p* < 0.02).

Assuming serum total 25-(OH)D levels in the range of 30–50 ng/mL to be the physiological norm 17.5% (*n* = 7) of athletes achieved this level. We found that 42.5% (*n* = 17) of the participants had a total 25-(OH)D concentration of below 20 ng/ml, which is defined as vitamin D deficiency (Table 1) (Pludowski et al., 2018).

There was no statistical difference in vitamin D metabolites [total 25-OH)D, 25-(OH)D_3_, 25-(OH)D_2_, 24,25-(OH)_2_D_3_, 3-epi-25-(OH)D_3_, 1,25-(OH)_2_D_3_], VDBP, free 25-(OH)D (M1, M2), and bioavailable 25-(OH)D between the indoor and outdoor player group (Table 1).

Handgrip strength (L) was higher in judoists (*p* = 0.018) than in football players. There were no changes observed between groups for handgrip strength (R), vertical jump parameters, and VO_2max_ (Table 1).

Multiple regression analyses demonstrated association between iPTH, Ca, ACa, and vitamin D metabolites, VDBP, free (M1, M2), and bioavailable 25-(OH)D in both groups with no intergroup differences.

We performed multiple regression analyses to explore possible association between vitamin D metabolites and the athletic performance parameters in indoor (Table 2) and outdoor players (Table 3). Furthermore, we performed multiple regression analysis also to determine relationship between VDBP, free (M1, M2), bioavailable 25-(OH)D, and the physical fitness tests for both groups (Table 4).

**TABLE 2:** Association of athletic performance with vitamin D metabolites by multiple regression analyses* in indoor players (*n* = 16).

Physical fitness test variables	Total 25-(OH)D (ng/mL)		24,25-(OH)_2_D_3_ (ng/mL)		3-epi-25-(OH)D_3_ (ng/mL)		1,25(OH)_2_D (pg/ml)	
	ß (95% CI)	*p*-value	ß (95% CI)	*p*-value	ß (95% CI)	*p*-value	ß (95% CI)	*p*-value
VO_2max_ (ml/kg/min)	0.194 (−0.447–0.835)	0.55	0.173 (−0.470–0.817)	0.60	0.050 (−0.602–0.703)	0.88	−0.373 (−0.980–0.233)	0.23
Handgrip strength L (kg)	0.456 (−0.010–0.923)	0.055	0.305 (−0.194–0.804)	0.23	0.615 (0.202–1.028)	**0.004**	−0.338 (−0.831–0.155)	0.18
Handgrip strength R (kg)	0.461 (−0.003–0.926)	0.052	0.415 (−0.061–0.892)	0.088	0.612 (0.198–1.026)	**0.004**	−0.480 (−0.939–−0.020)	**0.041**
Jump height (cm)	0.540 (0.099–0.980)	**0.016**	0.550 (0.113–0.990)	**0.014**	0.701 (0.327–1.074)	**<0.001**	−0.378 (−0.863–0.107)	0.13
Peak force (N)	0.287 (-0.214–0.789)	0.26	0.339 (-0.154–0.832)	0.18	0.460 (−0.005–0.925)	0.052	−0.339 (−0.832–0.153)	0.18
Peak velocity [m/s]	0.518 (0.070–0.970)	**0.024**	0.534 (0.092–0.980)	**0.018**	0.673 (0.286–1.060)	**<0.001**	−0.421 (−0.896–0.054)	0.083
Peak power (W)	0.538 (0.096–0.980)	**0.017**	0.536 (0.094–0.980)	**0.018**	0.766 (0.430–1.103)	**<0.001**	−0.393 (−0.875–0.089)	0.11
RSImod (m/s)	0.511 (0.060–0.960)	**0.026**	0.547 (0.108–0.990)	**0.015**	0.599 (0.179–1.018)	**0.005**	−0.302 (−0.802–0.197)	0.24
Peak force (N/kg)	0.153 (-0.365–0.670)	0.56	0.331 (-0.163–0.826)	0.19	0.108 (−0.413–0.628)	0.69	−0.339 (−0.832–0.154)	0.18
Peak power (W/kg)	0.681 (0.298–1.065)	**<0.001**	0.716 (0.350–1.081)	**<0.001**	0.784 (0.459–1.109)	**<0.001**	−0.477 (−0.938–−0.017)	**0.042**
Peak propulsive force/kg (N/kg)	0.183 (-0.331–0.698)	0.48	0.372 (-0.115–0.858)	0.13	0.119 (−0.401–0.639)	0.65	−0.353 (−0.843–0.137)	0.16
AVG propulsive force/kg (N/kg)	0.616 (0.203–1.029)	**0.003**	0.716 (0.350–1.082)	**<0.001**	0.530 (0.086–0.970)	**0.019**	−0.376 (−0.862–0.109)	0.13
Peak propulsive power/kg (W/kg)	0.686 (0.305–1.067)	**<0.001**	0.725 (0.364–1.086)	**<0.001**	0.776 (0.446–1.106)	**<0.001**	−0.482 (−0.941–−0.023)	**0.040**
AVG propulsive power/kg (W/kg)	0.631 (0.225–1.038)	**0.002**	0.719 (0.356–1.083)	**<0.001**	0.703 (0.331–1.076)	**<0.001**	−0.469 (−0.932–−0.007)	**0.047**

Statistically significant variables were adjusted for age, body mass, and body fat; ß, unstandardized coefficient; 95%CI, confidence interval; VDBP, vitamin D binding protein; VO_2max_, maximal oxygen uptake; L, left; R, right; CMJ, countermovement vertical jump; AVG, average. Bold values are statistically significant.

**TABLE 3:** Association of athletic performance with vitamin D metabolites by multiple regression analyses* in outdoor players (*n* = 24).

	Total 25-(OH)D [ng/mL]		24,25-(OH)_2_D_3_ [ng/mL]		3-epi-25-(OH)D_3_ [ng/mL]		1,25(OH)_2_D [pg/mL]	
Physical fitness test variables	ß (95% CI)	*p*-value	ß (95% CI)	*p*-value	ß (95% CI)	*p*-value	ß (95% CI)	*p*-value
VO_2max_ (ml/kg/min)	0.095 (−0.321–0.511)	0.66	0.232 (−0.174–0.638)	0.26	0.090 (−0.326–0.507)	0.67	−0.112 (−0.527–0.303)	0.60
Handgrip strength L (kg)	−0.049 (−0.466–0.369)	0.82	−0.062 (−0.479–0.355)	0.77	0.061 (−0.356–0.478)	0.78	−0.152 (−0.565–0.261)	0.47
Handgrip strength R (kg)	0.079 (−0.337–0.496)	0.71	0.113 (−0.302–0.528)	0.59	0.117 (−0.298–0.532)	0.58	−0.041 (−0.459–0.376)	0.85
Jump height (cm)	0.314 (−0.124–0.753)	0.16	0.265 (−0.180–0.711)	0.24	0.359 (−0.072–0.790)	0.10	−0.077 (−0.538–0.383)	0.74
Peak force (N)	0.339 (−0.096–0.774)	0.13	0.303 (−0.137–0.744)	0.18	0.444 (0.030–0.858)	**0.035**	−0.140 (−0.597–0.318)	0.55
Peak velocity (m/s)	0.276 (−0.168–0.720)	0.22	0.219 (−0.232–0.669)	0.34	0.331 (−0.105–0.767)	0.14	−0.087 (−0.548–0.373)	0.71
Peak power (W)	0.432 (0.015–0.848)	**0.042**	0.312 (−0.127–0.751)	0.16	0.492 (0.089–0.894)	**0.017**	−0.321 (−0.758–0.117)	0.15
RSImod (m/s)	0.264 (−0.182–0.709)	0.25	0.308 (−0.132–0.747)	0.17	0.229 (−0.221–0.678)	0.32	−0.058 (−0.519–0.403)	0.80
Peak force (N/kg)	0.118 (−0.341–0.577)	0.61	0.188 (−0.266–0.641)	0.42	0.311 (−0.128–0.750)	0.16	0.128 (−0.330–0.586)	0.58
Peak power (W/kg)	0.261 (−0.185–0.707)	0.25	0.203 (−0.250–0.655)	0.38	0.384 (−0.042–0.811)	0.08	−0.130 (−0.588–0.328)	0.58
Peak propulsive force/kg (N/kg)	0.127 (−0.331–0.585)	0.59	0.191 (−0.263–0.644)	0.41	0.323 (−0.115–0.760)	0.15	0.119 (−0.340–0.578)	0.61
AVG propulsive force/kg (N/kg)	0.337 (−0.098–0.772)	0.13	0.311 (-0.128–0.750)	0.17	0.475 (0.068–0.881)	**0.022**	-0.042 (-0.503–0.420)	0.86
Peak propulsive power/kg (W/kg)	0.274 (-0.170–0.718)	0.23	0.214 (-0.237–0.665)	0.35	0.395 (−0.030–0.819)	0.07	−0.142 (−0.599–0.316)	0.54
AVG propulsive power/kg (W/kg)	0.316 (−0.122–0.754)	0.16	0.260 (−0.186–0.706)	0.25	0.432 (0.016–0.849)	**0.042**	−0.029 (−0.491–0.433)	0.90

Statistically significant variables were adjusted for age, body mass, and body fat; ß, unstandardized coefficient; 95% CI, confidence interval; VDBP, vitamin D binding protein; VO_2max_, maximal oxygen uptake; L, left; R, right; CMJ, countermovement vertical jump; AVG, average. Bold values are statistically significant.

**TABLE 4:** Association of athletic performance with VDBP, free, and bioavailable 25-(OH)D concentration by multiple regression analyses* in indoor players (*n* = 16).

Physical fitness test variables	VDBP [µmol/L]		Free 25-(OH)D M1 [pg/mL]		Free 25-(OH)D M2 [pg/mL]		Bioavailable 25-(OH)D [ng/mL]	
	ß (95% CI)	*p*-value	ß (95% CI)	*p*-value	ß (95% CI)	*p*-value	ß (95% CI)	*p*-value
VO_2max_ (ml/kg/min)	0.144 (−0.502–−0.791)	0.66	0.066 (−0.585–−0.718)	0.84	0.066 (−0.586–−0.718)	0.84	0.093 (−0.557–−0.744)	0.78
Handgrip strength L (kg)	0.066 (−0.457–−0.589)	0.80	0.456 (−0.010–−0.922)	**0.05**	0.459 (−0.007–−0.924)	**0.05**	0.427 (−0.047–−0.901)	0.077
Handgrip strength R (kg)	0.154 (−0.363–−0.672)	0.56	0.425 (−0.050–−0.899)	0.079	0.429 (−0.044–−0.903)	0.075	0.404 (−0.075–−0.883)	0.10
Jump height (cm)	−0.259 (−0.765–−0.247)	0.32	0.668 (0.278–−1.058)	**<0.001**	0.670 (0.280–−1.059)	**<0.001**	0.635 (0.230–−1.039)	**0.002**
Peak force (N)	0.274 (−0.230–−0.778)	0.29	0.227 (−0.283–−0.737)	0.38	0.235 (−0.274–−0.744)	0.37	0.179 (−0.336–−0.695)	0.50
Peak velocity (m/s)	−0.230 (−0.740–−0.280)	0.38	0.633 (0.227–−1.038)	**0.002**	0.635 (0.230–−1.040)	**0.002**	0.596 (0.176–−1.017)	**0.005**
Peak power (W)	0.091 (−0.431–−0.613)	0.73	0.562 (0.128–−0.995)	**0.011**	0.568 (0.137–−0.999)	**0.010**	0.524 (0.077–−0.97)	**0.021**
RSImod (m/s)	−0.216 (−0.728–−0.295)	0.41	0.580 (0.154–−1.007)	**0.008**	0.581 (0.154–−1.007)	**0.008**	0.552 (0.115–−0.99)	**0.013**
Peak force (N/kg)	−0.021 (−0.545–−0.503)	0.94	0.131 (−0.388–−0.650)	0.62	0.133 (−0.386–−0.653)	0.61	0.098 (−0.424–−0.619)	0.71
Peak power (W/kg)	−0.183 (−0.698–−0.332)	0.49	0.773 (0.440–−1.105)	**<0.001**	0.775 (0.444–−1.106)	**<0.001**	0.750 (0.403–−1.096)	**<0.001**
Peak propulsive force/kg (N/kg)	−0.018 (−0.541–−0.506)	0.95	0.159 (−0.358–−0.676)	0.55	0.162 (−0.355–−0.679)	0.54	0.126 (−0.393–−0.646)	0.63
AVG propulsive force/kg (N/kg)	−0.042 (−0.565–−0.481)	0.88	0.612 (0.198–−1.026)	**0.004**	0.614 (0.200–−1.027)	**0.004**	0.602 (0.184–−1.020)	**0.005**
Peak propulsive power/kg (W/kg)	−0.170 (−0.686–−0.346)	0.52	0.773 (0.440–−1.105)	**<0.001**	0.775 (0.444–−1.106)	**<0.001**	0.751 (0.405–−1.097)	**<0.001**
AVG propulsive power/kg (W/kg)	−0.195 (−0.709–−0.319)	0.46	0.713 (0.346–−1.080)	**<0.001**	0.716 (0.350–−1.082)	**<0.001**	0.683 (0.300–−1.065)	**<0.001**

*Statistically significant variables were adjusted for age, body mass, and body fat; ß, unstandardized coefficient; 95% CI, confidence interval; VDBP, vitamin D binding protein; VO_2max_, maximal oxygen uptake; L, left; R, right; CMJ, countermovement vertical jump; AVG, average; M1, equations by Bikle et al. (1986); M2, equations by Vermeulen et al. (1999). Bold values are statistically significant.

Table 2 presents the association of vitamin D metabolites with athletic performance by multiple regression analyses in indoor players. Multiple regression analysis demonstrated that total 25-(OH)D, 24,25-(OH)_2_D_3_, and 3-epi-25-(OH)D_3_ were significantly associated with jump height, peak velocity, peak power, RSImod, propulsive force, and power_._ There was also significant correlation between 3-epi-25(OH)D_3_ and handgrip strength (L, R). Multiple regression analysis indicated that 1,25(OH)_2_D significantly associated with handgrip strength (R), peak power, and propulsive power.

There was significant correlation between free 25-(OH)D (M1, M2) and handgrip strength (L) in indoor players. Analysis showed that free 25-(OH)D (M1,M2) and bioavailable 25-(OH)D significantly associated with jump height, peak velocity, power, RSImod, propulsive force, and power (Table 4).

In the group of outdoor players, multiple regression analysis showed that peak power was significantly associated with serum total 25-(OH)D and 3-epi-25-(OH)D_3_. The association between peak force, propulsive force and power, and 3-epi-25-(OH)D_3_ was observed (Table 3).

Table 5 presents the association of VDBP, free (M1, M2), and bioavailable 25-(OH)D with athletic performance by multiple regression analyses in outdoor players. According to our results, there was no significant correlation between VDBP, free (M1, M2), bioavailable 25-(OH)D, and physical performance parameters in outdoor athletes.

**TABLE 5:** Association of athletic performance with VDBP, free, and bioavailable 25-(OH)D concentration by multiple regression analyses* in outdoor players (*n* = 24).

Physical fitness test variables	VDBP [µmol/L]		Free 25-(OH)D M1 [pg/mL]		Free 25-(OH)D M2 [pg/mL]		Bioavailable 25-(OH)D [ng/mL]	
	ß (95% CI)	*p*-value	ß (95% CI)	*p*-value	ß (95% CI)	*p*-value	ß (95% CI)	*p*-value
VO_2max_ (ml/kg/min)	−0.141 (−0.565–−0.282)	0.51	0.073 (−0.353–−0.500)	0.74	0.072 (−0.354–−0.499)	0.74	0.069 (−0.358–−0.496)	0.75
Handgrip strength L (kg)	−0.282 (−0.692–−0.129)	0.18	0.135 (−0.289–−0.559)	0.53	0.131 (−0.293–−0.555)	0.54	0.141 (−0.283–−0.564)	0.51
Handgrip strength R (kg)	0.021 (−0.407–−0.448)	0.92	0.079 (−0.348–−0.505)	0.72	0.079 (−0.348–−0.505)	0.72	0.096 (−0.330–−0.521)	0.66
Jump height (cm)	0.160 (−0.309–−0.629)	0.50	0.201 (−0.265–−0.666)	0.40	0.203 (−0.263–−0.668)	0.39	0.185 (−0.282–−0.652)	0.44
Peak force (N)	−0.133 (−0.604–−0.338)	0.58	0.289 (−0.166–−0.744)	0.21	0.289 (−0.166–−0.744)	0.21	0.246 (−0.214–−0.707)	0.29
Peak velocity (m/s)	0.158 (−0.311–−0.628)	0.51	0.186 (−0.281–−0.653)	0.44	0.187 (−0.280–−0.654)	0.43	0.171 (−0.298–−0.639)	0.48
Peak power (W)	−0.064 (−0.539–−0.410)	0.79	0.356 (−0.089–−0.800)	0.12	0.356 (−0.089–−0.800)	0.12	0.319 (−0.132–−0.769)	0.17
RSImod (m/s)	−0.038 (−0.513–−0.437)	0.88	0.232 (−0.231–−0.694)	0.33	0.233 (−0.229–−0.695)	0.32	0.238 (−0.224–−0.700)	0.31
Peak force (N/kg)	−0.016 (−0.492–−0.459)	0.95	0.089 (−0.385–−0.562)	0.71	0.090 (−0.383–−0.564)	0.71	0.088 (−0.386–−0.561)	0.72
Peak power (W/kg)	0.021 (−0.455–−0.496)	0.93	0.222 (−0.241–−0.686)	0.35	0.223 (−0.240–−0.686)	0.35	0.217 (−0.247–−0.681)	0.36
Peak propulsive force/kg [N/kg]	−0.040 (−0.515–−0.435)	0.87	0.106 (−0.367–−0.578)	0.66	0.107 (−0.365–−0.580)	0.66	0.104 (−0.369–−0.577)	0.67
AVG propulsive force/kg [N/kg]	−0.087 (−0.561–−0.386)	0.72	0.298 (−0.155–−0.752)	0.20	0.299 (−0.154–−0.753)	0.20	0.303 (−0.150–−0.756)	0.19
Peak propulsive power/kg (W/kg)	−0.141 (−0.565–−0.282)	0.96	0.235 (−0.227–−0.697)	0.32	0.236 (−0.226–−0.698)	0.32	0.231 (−0.231–−0.694)	0.33
AVG propulsive power/kg (W/kg)	−0.282 (−0.692–−0.129)	0.82	0.231 (−0.232–−0.693)	0.33	0.233 (−0.230–−0.695)	0.32	0.224 (−0.239–−0.687)	0.34

Statistically significant variables were adjusted for age, body mass, and body fat; ß, unstandardized coefficient; 95% CI, confidence interval; VDBP, vitamin D binding protein; VO_2_max, maximal oxygen uptake; L, left; R, right; CMJ, countermovement vertical jump; AVG, average; M1, equations by Bikle et al. (1986); M2, equations by Vermeulen et al. (1999).

## 4 Discussion

This study represents a picture of the entire set of vitamin D metabolites in athletes. In this study, the enrolled athletes were classified into two groups, train indoor and outdoor. The aim of our study was to determine the relationship between metabolites of vitamin D, VDBP, free, bioavailable 25-(OH)D, related blood parameters (iPTH, Ca, and ACa), and physical fitness tests in athletes.

Physical activity can affect vitamin D metabolism and, hence, the final level of 25-(OH)D, main indicator of vitamin D status, and the other metabolites, all having important physiological roles. Further, it is possible that vitamin D metabolism is differently affected by different kinds of physical activity (in terms of muscle action and sun exposure). Thereby, it is interesting to compare these two situations.

Skeletal muscles express, other than the VDR, CYP27B1 (the gene encoding for 1alpha-hydroxylase, the enzyme that hydroxylates 25-(OH)D into 1,25-(OH)_2_D. Therefore, skeletal muscles are able to activate vitamin D (Latham et al., 2021). Although the biological relevance of the expression of this enzymatic activity is not well defined, it has been hypothesized that it is related to mitochondrial function and muscle regeneration, at least in rodents (Latham et al., 2021). Further, 1alpha-hydroxylase expression is decreased in denervation, in rats (Mori et al., 2020), while it is increased in muscle biopsies from amyotrophic lateral sclerosis patients (Si et al., 2020). Therefore, although the unclear mechanisms underlying its regulation, evidences suggest a direct connection between the muscle activity and vitamin D metabolism.

We showed that in the group of indoor judoists there was a significant correlation between total 25-(OH)D and vertical jump parameters (jump height, peak velocity, peak power, RSImod, and propulsive force and power). The relationship between total 25-(OH)D and physical performance levels of male athletes has been described in several studies (Fitzgerald et al., 2015; Kim et al., 2020; Most et al., 2021). Our study also showed the relationship between 24,25-(OH)_2_D_3_, 3-epi-25-(OH)D_3_, 1,25-(OH)_2_D, and vertical jump variables in indoor players. A small number of studies have assessed correlations between serum concentrations of 1,25-(OH)_2_D. Other vitamin D metabolites such as 24,25-(OH)_2_D_3_ and 3-epi-25-(OH)D_3_ have not been previously studied in relation to physical performance in athletes. Hassan-Smith et al. (2017) observed a statistically significant association between 1,25-(OH)_2_D and jump height-peak power in a group of 116 healthy human volunteers (79 women and 37 men; aged 20 ± 74 years). The authors demonstrated that 25-(OH)D_3_ and 24,25-(OH)_2_D_3_ were associated with efficiency (a measure of the relationship between maximum jump force and power, with the less force required to generate the same power, the more efficient the jump) in the studied group (Hassan-Smith et al., 2017). 24,25-(OH)_2_D_3_ is not considered to be physiologically active although studies involving animal models demonstrated that it plays an important role in normal bone integrity, function, and healing (Seo et al., 1997; Nemere et al., 2006).

We showed that in the group of outdoor players there was a significant association between serum total 25-(OH)D and peak power. Other studies demonstrated a positive association between 25-(OH)D levels and jump height in male soccer players (Koundourakis et al., 2014) and university-level outdoor athletes (Wilson-Barnes et al., 2020). We also showed the association between 3-epi-25-(OH)D_3_ and peak power, force, propulsive force, and power in outdoor athletes. Mieszkowski et al. (2020) demonstrated that serum 25-(OH)D_3_, 24,25-(OH)_2_D_3_, and 3-epi-25-(OH)D_3_ levels significantly increased after the ultra-marathon in control and supplemented group. C-3 epimerization is a common metabolic pathway of major metabolites of vitamin D_3_. 25-(OH)D_3_ undergoes epimerization, and 3-epi-25-(OH)D_3_ is the most prevalent form (Singh et al., 2006). The biological function of 3-epi-25-(OH)D_3_ is not well understood. To the best of our knowledge, this is the first paper reporting relationship between physical performance indicators and 24,25-(OH)_2_D_3_, 3-epi-25-(OH)D_3_ levels in athletes. Therefore, it is pivotal to study the effects of vitamin D metabolites’ action on skeletal muscle function in relation to athletic performance.

The free hormone hypothesis postulates that only the non-bound fraction (the free fraction) of hormones, which otherwise circulate in blood bound to their carrier proteins, is able to enter the cells and to exert their biologic effects (Bikle and Schwartz, 2019). Studies suggest that some functions of vitamin D may be more closely related to the free or bioavailable fraction of vitamin D than to total serum 25-(OH)D concentrations (Bikle et al., 2017; Owens et al., 2018).

Therefore, we considered whether the free fraction of 25-(OH)D correlates with musculoskeletal functions in relation to physical performance in athletes. To the best of our knowledge, there are no studies designed to assess VDBP, free, and bioavailable vitamin D status in athletes and its correlation to athletic performance. The impact of vitamin D on muscle strength, especially lower muscle strength in athletes, was evident in most studies (Hamilton et al., 2014; Koundourakis et al., 2014; Książek et al., 2016; Książek et al., 2018) and was physiologically explained by different vitamin D receptor expressions in various muscle groups (Bischoff et al., 2001; Hassan-Smith et al., 2017). Vitamin D affects the number and diameter of type II muscle fibers, which mainly regulate the ability to perform short high-power exercises (Dzik and Kaczor, 2019). Therefore, we used a vertical jump to check athletic performance in studied groups. A vertical jump is one of the essential motor skills that requires complex motor coordination, and it has been identified as one of the fundamental movement skills. Therefore, vertical jump test is used to evaluate simple lower limb muscular strength and complex tasks, such as sprint deceleration, sprint acceleration, throwing, and change of direction (Petrigna et al., 2019), which reflect physical effort during judo and football match.

We demonstrated associations between free and bioavailable 25-(OH)D levels and aspects of physical performance (handgrip strength L, vertical jump variables); this was, however, only observed within the indoor group. The differences in the obtained result between indoor and outdoor athletes may be due to the baseline individual training profile, different kinds of physical effort related to sport discipline, and hence activation of different muscle groups. This might explain the lack of significant association between VDBP, free, bioavailable 25-(OH)D, and physical performance variables in our outdoor players.

As a homeostatic perturbation, exercise and training affects calcium metabolism. Thereby, the measurement of PTH and calcium is relevant to assess the athlete’s health status as well as to contextualize the change observed in vitamin D metabolites (Lombardi et al., 2020). The results of our study showed that iPTH, Ca, and ACa were associated with and vitamin D metabolites, VDBP, free and bioavailable 25-(OH)D in both groups. However, we did not find differences in these associations between indoor and outdoor athletes. However, studies in this field are limited. In a recent study conducted at the British university athletes, we compared the seasonal effects (fall vs spring) of discipline, specific training, either indoors or outdoors on bone indices, and vitamin D status. Seasonal variation of serum 25-(OH)D was found independent from training modality (indoor vs outdoor) and PTH resulted negatively associated with the vitamin D status in the combined groups, indoor group, and more significant in the outdoor group during the spring term (Wilson-Barnes et al., 2020). In military recruits, a 32-week training program reduced vitamin D status (25-(OH)D < 50 nmol/L) and enhanced concentrations of PTH associated with the occurrence of stress fractures (Davey et al., 2016). Similarly to our study, instead, PTH resulted unaffected by an 8-week repeated sprint training regimen in young active men (Sansoni et al., 2018) and by 13 weeks of military training (O'Leary et al., 2019), despite the changes in bone indexes.

### 4.1 Limitations and Strengths of the Study

Some limitations should be considered when evaluating the result of this analysis: first, the limited sample size, so further research on a larger group of athletes is needed, and second, there was a lack of a non-sportive control group. Furthermore, our data are associational and no causal links between the vitamin D metabolites and physical performance can be drawn from this study. A notable strength of our investigation is, instead, the homogeneity within each cohort of experienced male athletes sharing the same age and anthropometry. We performed blood drawing and physical performance test in the same period (within two weeks) for both cohorts and strong pre-analytical phase.

## 5 Conclusion

Our study showed a significant correlation between vitamin D metabolites [total 25-(OH)D, 24,25-(OH)_2_D_3_, 3-epi-25-(OH)D_3_, and 1,25-(OH)_2_D] and handgrip strength and vertical jump variables in indoor players. It also showed a significant association between 3-epi-25-(OH)D_3_ and vertical jump parameters in outdoor players. The results of our study demonstrated the relationship between free, bioavailable 25-(OH)D, and vertical jump variables only in the group of indoor players.

In conclusion, this is the first study demonstrating the relationship between vitamin D metabolites, free and bioavailable 25-(OH)D, and physical performance in athletes. These observations imply that vitamin D metabolites might be involved in skeletal muscle function. However, more work is needed to explore the role of vitamin D metabolites in relation to athletic performance.

## Data Availability

The raw data supporting the conclusion of this article will be made available by the authors, without undue reservation.
