# Corrigendum: Relationship between metabolites of vitamin D, free 25-(OH)D, and physical performance in indoor and outdoor athletes

**DOI:** 10.3389/fphys.2022.1003648

**Published:** 2022-08-26

**Authors:** Anna Książek, Aleksandra Zagrodna, Małgorzata Słowińska-Lisowska, Giovanni Lombardi

**Affiliations:** ^1^ Department of Biological and Medical Basis of Sport, Faculty of Physical Education and Sports, Wroclaw University of Health and Sport Sciences, Wroclaw, Poland; ^2^ Laboratory of Experimental Biochemistry & Molecular Biology, I.R.C.C.S. Istituto Ortopedico Galeazzi, Milano, Italy; ^3^ Department of Athletics, Strength and Conditioning, Poznań University of Physical Education, Poznań, Poland

**Keywords:** vitamin D, 24,25-(OH)2D3, 3-epi-25-(OH)D, 1,25(OH)2D, VDBP, vertical jump, physical performance

In the published article, there was an error regarding the affiliation(s) for the author, **Giovanni Lombardi**. As well as having **affiliation** 2, the author should also have “Department of Athletics, Strength and Conditioning, Poznań University of Physical Education, Poznań, Poland.”

The authors apologize for this error and state that this does not change the scientific conclusions of the article in any way. The original article has been updated.

